# Erratum

**DOI:** 10.1590/1518-8345.0000.3038

**Published:** 2018-10-08

Regarding the article “Jacobson and Truax Method: evaluation of the clinical effectiveness of a home care program after prostatectomy”, with DOI number: 1518-8345.2249.3003, published in Rev. Latino-Am. Enfermagem, 2018;26:e3003, page 1:

Where was written:

“Enfermagem Fundamental, Escola de Enfermagem da Universidade de São Paulo, Ribeirão Preto, SP, Brazil.”

Now Read:

“Escola de Enfermagem de Ribeirão Preto, Universidade de São Paulo, PAHO/WHO Collaborating Centre for Nursing Research Development, Ribeirão Preto, SP, Brazil.”

Regarding the article “Coping strategies of people living with AIDS in face of the disease”, with DOI number: 1518-8345.2284.2985, published in Rev. Latino-Am. Enfermagem, 2018;26:e2985, page 1:

Where was written:

“^3^ PhD, RN, Serviço de Atendimento Móvel de Urgência, Prefeitura Municipal de Timon, Timon, MA, Brazil. Adjunct Professor, Departamento de Enfermagem, Universidade Federal de Sergipe, Lagarto, SE, Brazil.

^4^ MSc, Professor, Departamento de Enfermagem, Universidade Federal do Rio Grande do Norte, Natal, RN, Brazil.

^5^ Doctoral Student, Universidade Federal do Rio Grande do Norte, Natal, RN, Brazil.

^6^ Doctoral Student, Programa de Pós-Graduação em Enfermagem, Universidade Federal do Rio Grande do Norte, Natal, RN, Brazil. RN, Maternidade Professor Leide Morais, Natal, RN, Brazil. Professor, Departamento de Enfermagem, Universidade Federal do Rio Grande do Norte, Natal, RN, Brazil.”

Now Read:

“^3^ PhD, Adjunct Professor, Departamento de Enfermagem, Universidade Federal de Sergipe, Lagarto, SE, Brazil.

^4^ MSc, Substitute Professor, Departamento de Enfermagem, Universidade Federal do Rio Grande do Norte, Natal, RN, Brazil.

^5^ Doctoral Student, Departamento de Enfermagem, Universidade Federal do Rio Grande do Norte, Natal, RN, Brazil. Substitute Professor, Departamento de Enfermagem, Universidade Federal do Rio Grande do Norte, Natal, RN, Brazil.

^6^ Doctoral Student, Departamento de Enfermagem, Universidade Federal do Rio Grande do Norte, Natal, RN, Brazil. RN, Maternidade Professor Leide Morais, Natal, RN, Brazil.”

